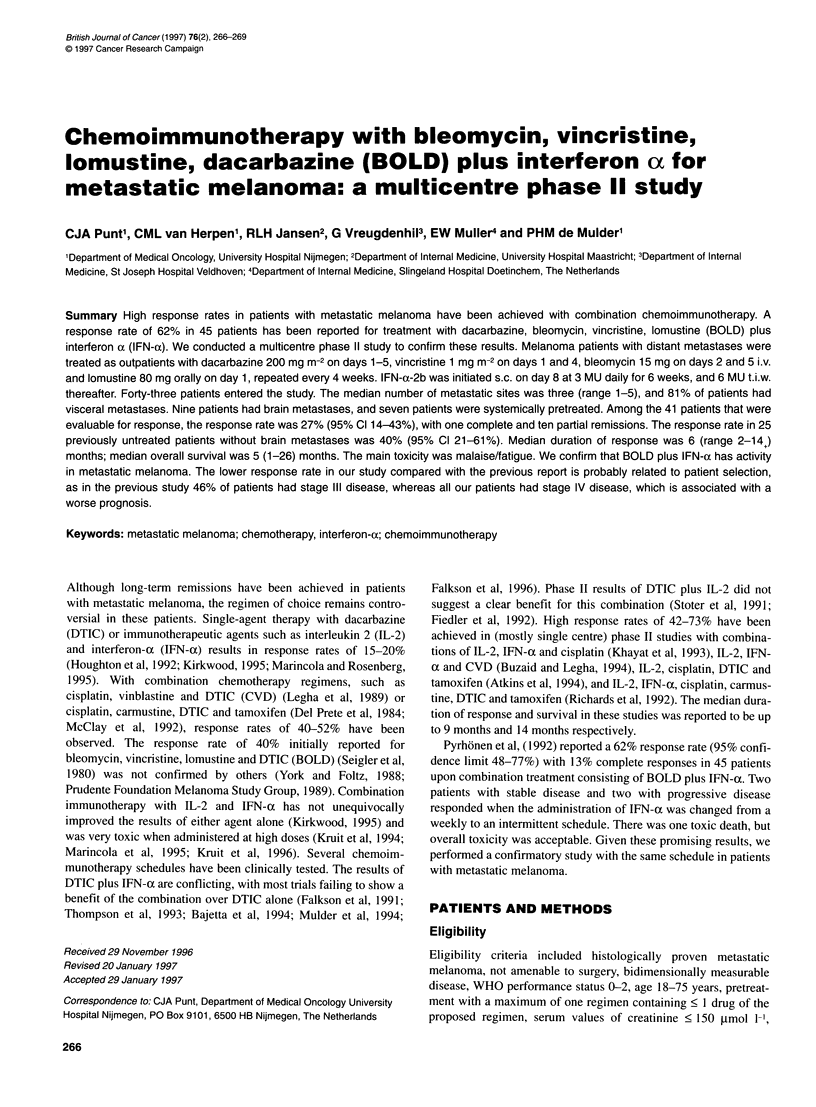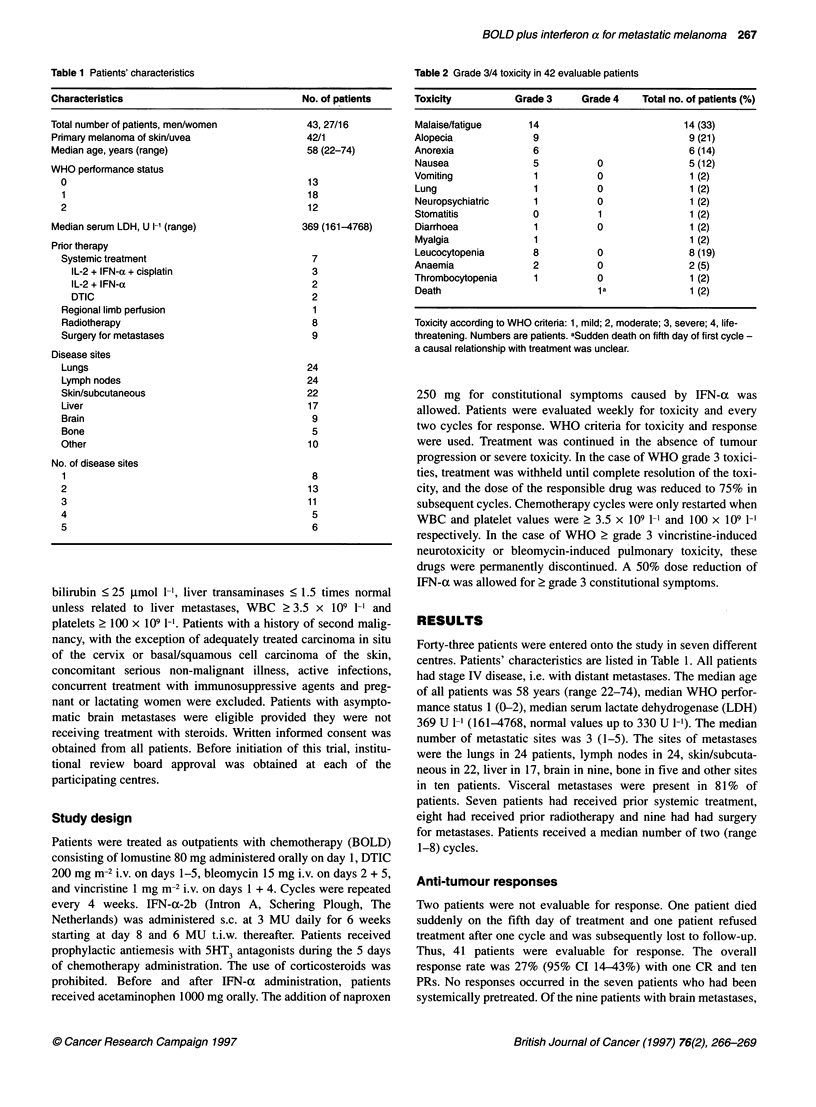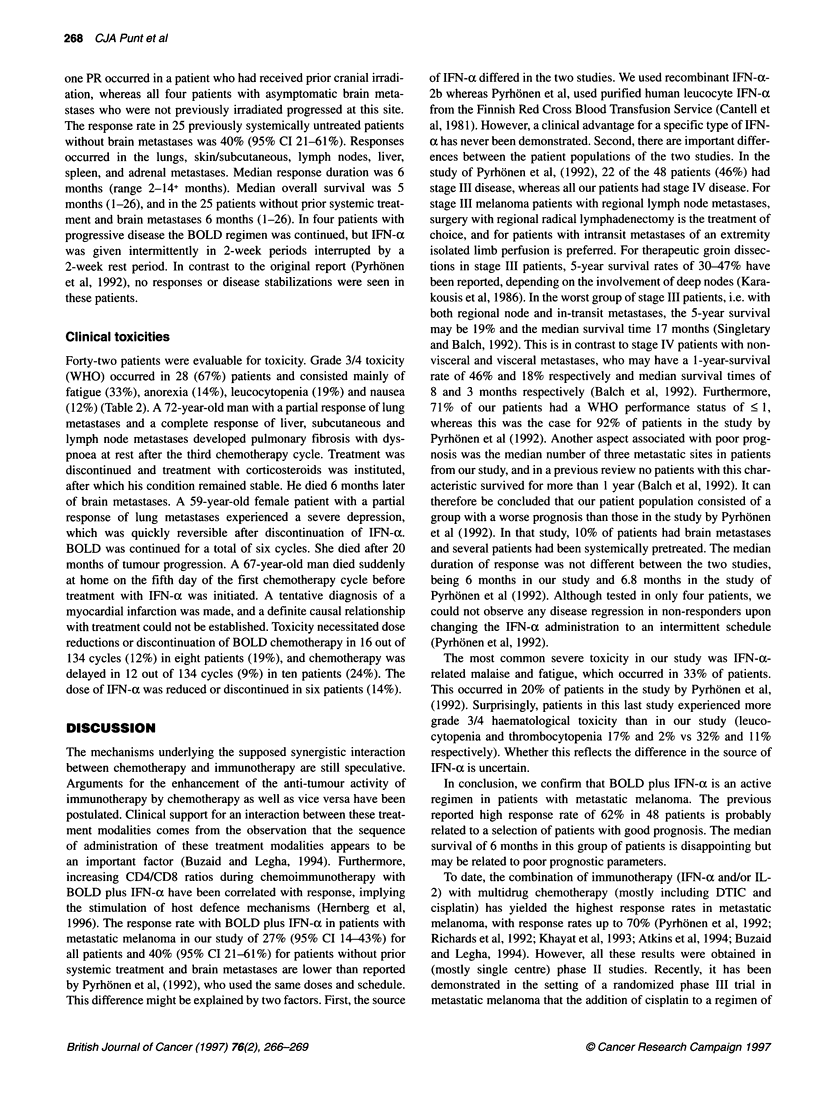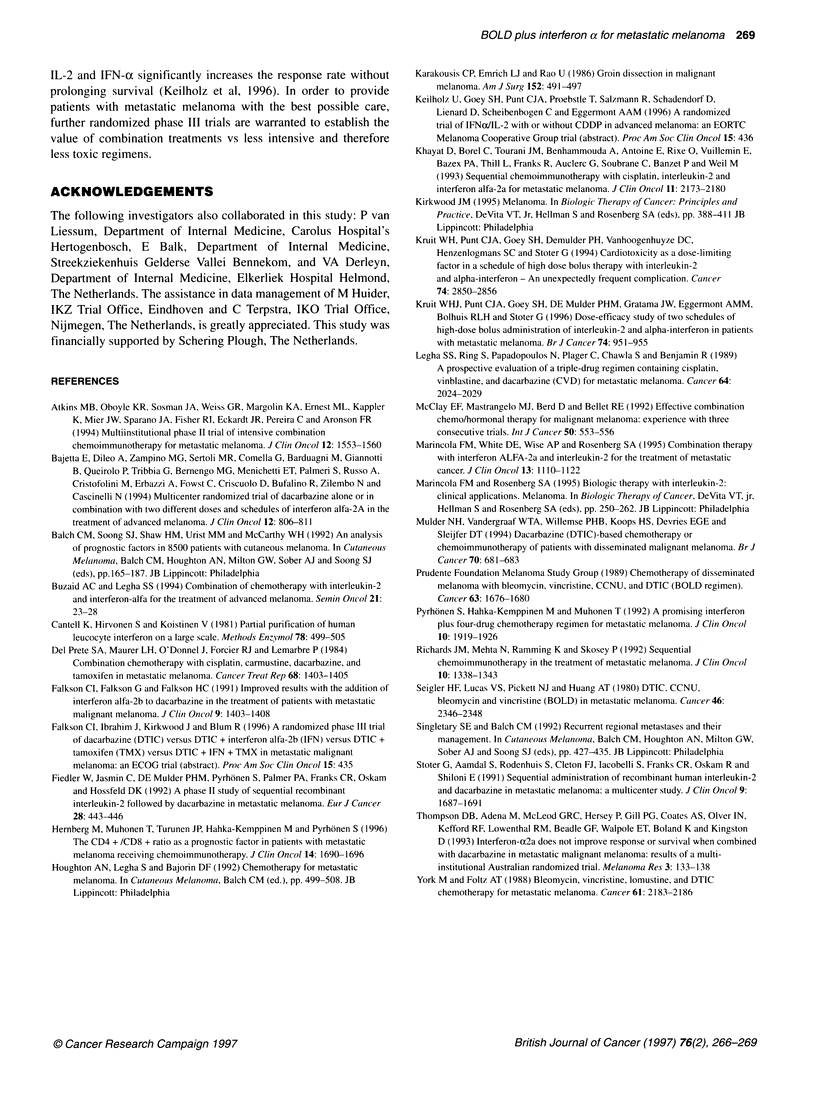# Chemoimmunotherapy with bleomycin, vincristine, lomustine, dacarbazine (BOLD) plus interferon alpha for metastatic melanoma: a multicentre phase II study.

**DOI:** 10.1038/bjc.1997.374

**Published:** 1997

**Authors:** C. J. Punt, C. M. van Herpen, R. L. Jansen, G. Vreugdenhil, E. W. Muller, P. H. de Mulder

**Affiliations:** Department of Medical Oncology, University Hospital Nijmegen, The Netherlands.

## Abstract

High response rates in patients with metastatic melanoma have been achieved with combination chemoimmunotherapy. A response rate of 62% in 45 patients has been reported for treatment with dacarbazine, bleomycin, vincristine, lomustine (BOLD) plus interferon alpha (IFN-alpha). We conducted a multicentre phase II study to confirm these results. Melanoma patients with distant metastases were treated as outpatients with dacarbazine 200 mg m(-2) on days 1-5, vincristine 1 mg m(-2) on days 1 and 4, bleomycin 15 mg on days 2 and 5 i.v. and lomustine 80 mg orally on day 1, repeated every 4 weeks. IFN-alpha-2b was initiated s.c. on day 8 at 3 MU daily for 6 weeks, and 6 MU t.i.w. thereafter. Forty-three patients entered the study. The median number of metastatic sites was three (range 1-5), and 81% of patients had visceral metastases. Nine patients had brain metastases, and seven patients were systemically pretreated. Among the 41 patients that were evaluable for response, the response rate was 27% (95% CI 14-3%), with one complete and ten partial remissions. The response rate in 25 previously untreated patients without brain metastases was 40% (95% CI 21-61%). Median duration of response was 6 (range 2-14+) months; median overall survival was 5 (1-26) months. The main toxicity was malaise/fatigue. We confirm that BOLD plus IFN-alpha has activity in metastatic melanoma. The lower response rate in our study compared with the previous report is probably related to patient selection, as in the previous study 46% of patients had stage III disease, whereas all our patients had stage IV disease, which is associated with a worse prognosis.


British Journal of Cancer (1997) 76(2), 266–269
© 1997 Cancer Research Campaign

### Chemoimmunotherapy with bleomycin, vincristine, lomustine, dacarbazine (BOLD) plus interferon α for metastatic melanoma: a multicentre phase II study

**CJA Punt[1], CML van Herpen[1], RLH Jansen[2], G Vreugdenhil[3], EW Muller[4] and PHM de Mulder[1]**

[1]Department of Medical Oncology, University Hospital Nijmegen; [2]Department of Internal Medicine, University Hospital Maastricht; [3]Department of Internal Medicine, St Joseph Hospital Veldhoven; [4]Department of Internal Medicine, Slingeland Hospital Doetinchem, The Netherlands

**Summary** High response rates in patients with metastatic melanoma have been achieved with combination chemoimmunotherapy. A response rate of 62% in 45 patients has been reported for treatment with dacarbazine, bleomycin, vincristine, lomustine (BOLD) plus interferon α (IFN-α). We conducted a multicentre phase II study to confirm these results. Melanoma patients with distant metastases were treated as outpatients with dacarbazine 200 mg $m^{-2}$ on days 1–5, vincristine 1 mg $m^{-2}$ on days 1 and 4, bleomycin 15 mg on days 2 and 5 i.v. and lomustine 80 mg orally on day 1, repeated every 4 weeks. IFN-α-2b was initiated s.c. on day 8 at 3 MU daily for 6 weeks, and 6 MU t.i.w. thereafter. Forty-three patients entered the study. The median number of metastatic sites was three (range 1–5), and 81% of patients had visceral metastases. Nine patients had brain metastases, and seven patients were systemically pretreated. Among the 41 patients that were evaluable for response, the response rate was 27% (95% CI 14–43%), with one complete and ten partial remissions. The response rate in 25 previously untreated patients without brain metastases was 40% (95% CI 21–61%). Median duration of response was 6 (range 2–$14_+$) months; median overall survival was 5 (1–26) months. The main toxicity was malaise/fatigue. We confirm that BOLD plus IFN-α has activity in metastatic melanoma. The lower response rate in our study compared with the previous report is probably related to patient selection, as in the previous study 46% of patients had stage III disease, whereas all our patients had stage IV disease, which is associated with a worse prognosis.

**Keywords:** metastatic melanoma; chemotherapy, interferon-α; chemoimmunotherapy

Although long-term remissions have been achieved in patients with metastatic melanoma, the regimen of choice remains controversial in these patients. Single-agent therapy with dacarbazine (DTIC) or immunotherapeutic agents such as interleukin 2 (IL-2) and interferon-α (IFN-α) results in response rates of 15–20% (Houghton et al, 1992; Kirkwood, 1995; Marincola and Rosenberg, 1995). With combination chemotherapy regimens, such as cisplatin, vinblastine and DTIC (CVD) (Legha et al, 1989) or cisplatin, carmustine, DTIC and tamoxifen (Del Prete et al, 1984; McClay et al, 1992), response rates of 40–52% have been observed. The response rate of 40% initially reported for bleomycin, vincristine, lomustine and DTIC (BOLD) (Seigler et al, 1980) was not confirmed by others (York and Foltz, 1988; Prudente Foundation Melanoma Study Group, 1989). Combination immunotherapy with IL-2 and IFN-α has not unequivocally improved the results of either agent alone (Kirkwood, 1995) and was very toxic when administered at high doses (Kruit et al, 1994; Marincola et al, 1995; Kruit et al, 1996). Several chemoimmunotherapy schedules have been clinically tested. The results of DTIC plus IFN-α are conflicting, with most trials failing to show a benefit of the combination over DTIC alone (Falkson et al, 1991; Thompson et al, 1993; Bajetta et al, 1994; Mulder et al, 1994; Falkson et al, 1996). Phase II results of DTIC plus IL-2 did not suggest a clear benefit for this combination (Stoter et al, 1991; Fiedler et al, 1992). High response rates of 42–73% have been achieved in (mostly single centre) phase II studies with combinations of IL-2, IFN-α and cisplatin (Khayat et al, 1993), IL-2, IFN-α and CVD (Buzaid and Legha, 1994), IL-2, cisplatin, DTIC and tamoxifen (Atkins et al, 1994), and IL-2, IFN-α, cisplatin, carmustine, DTIC and tamoxifen (Richards et al, 1992). The median duration of response and survival in these studies was reported to be up to 9 months and 14 months respectively.

Pyrhönen et al, (1992) reported a 62% response rate (95% confidence limit 48–77%) with 13% complete responses in 45 patients upon combination treatment consisting of BOLD plus IFN-α. Two patients with stable disease and two with progressive disease responded when the administration of IFN-α was changed from a weekly to an intermittent schedule. There was one toxic death, but overall toxicity was acceptable. Given these promising results, we performed a confirmatory study with the same schedule in patients with metastatic melanoma.

*Received 29 November 1996*
*Revised 20 January 1997*
*Accepted 29 January 1997*

*Correspondence to:* CJA Punt, Department of Medical Oncology University Hospital Nijmegen, PO Box 9101, 6500 HB Nijmegen, The Netherlands

### PATIENTS AND METHODS

### Eligibility

Eligibility criteria included histologically proven metastatic melanoma, not amenable to surgery, bidimensionally measurable disease, WHO performance status 0–2, age 18–75 years, pretreatment with a maximum of one regimen containing ≤ 1 drug of the proposed regimen, serum values of creatinine ≤ 150 μmol $l^{-1}$,

**Table 1** Patients' characteristics

| Characteristics | No. of patients |
|---|---|
| Total number of patients, men/women | 43, 27/16 |
| Primary melanoma of skin/uvea | 42/1 |
| Median age, years (range) | 58 (22–74) |
| WHO performance status | |
| 0 | 13 |
| 1 | 18 |
| 2 | 12 |
| Median serum LDH, U $l^{-1}$ (range) | 369 (161–4768) |
| Prior therapy | |
| Systemic treatment | 7 |
| IL-2 + IFN-α + cisplatin | 3 |
| IL-2 + IFN-α | 2 |
| DTIC | 2 |
| Regional limb perfusion | 1 |
| Radiotherapy | 8 |
| Surgery for metastases | 9 |
| Disease sites | |
| Lungs | 24 |
| Lymph nodes | 24 |
| Skin/subcutaneous | 22 |
| Liver | 17 |
| Brain | 9 |
| Bone | 5 |
| Other | 10 |
| No. of disease sites | |
| 1 | 8 |
| 2 | 13 |
| 3 | 11 |
| 4 | 5 |
| 5 | 6 |

bilirubin ≤ 25 μmol $l^{-1}$, liver transaminases ≤ 1.5 times normal unless related to liver metastases, WBC ≥ 3.5 × $10^9$ $l^{-1}$ and platelets ≥ 100 × $10^9$ $l^{-1}$. Patients with a history of second malignancy, with the exception of adequately treated carcinoma in situ of the cervix or basal/squamous cell carcinoma of the skin, concomitant serious non-malignant illness, active infections, concurrent treatment with immunosuppressive agents and pregnant or lactating women were excluded. Patients with asymptomatic brain metastases were eligible provided they were not receiving treatment with steroids. Written informed consent was obtained from all patients. Before initiation of this trial, institutional review board approval was obtained at each of the participating centres.

### Study design

Patients were treated as outpatients with chemotherapy (BOLD) consisting of lomustine 80 mg administered orally on day 1, DTIC 200 mg $m^{-2}$ i.v. on days 1–5, bleomycin 15 mg i.v. on days 2 + 5, and vincristine 1 mg $m^{-2}$ i.v. on days 1 + 4. Cycles were repeated every 4 weeks. IFN-α-2b (Intron A, Schering Plough, The Netherlands) was administered s.c. at 3 MU daily for 6 weeks starting at day 8 and 6 MU t.i.w. thereafter. Patients received prophylactic antiemesis with $5HT_3$ antagonists during the 5 days of chemotherapy administration. The use of corticosteroids was prohibited. Before and after IFN-α administration, patients received acetaminophen 1000 mg orally. The addition of naproxen

**Table 2** Grade 3/4 toxicity in 42 evaluable patients

| Toxicity | Grade 3 | Grade 4 | Total no. of patients (%) |
|---|---|---|---|
| Malaise/fatigue | 14 | | 14 (33) |
| Alopecia | 9 | | 9 (21) |
| Anorexia | 6 | | 6 (14) |
| Nausea | 5 | 0 | 5 (12) |
| Vomiting | 1 | 0 | 1 (2) |
| Lung | 1 | 0 | 1 (2) |
| Neuropsychiatric | 1 | 0 | 1 (2) |
| Stomatitis | 0 | 1 | 1 (2) |
| Diarrhoea | 1 | 0 | 1 (2) |
| Myalgia | 1 | | 1 (2) |
| Leucocytopenia | 8 | 0 | 8 (19) |
| Anaemia | 2 | 0 | 2 (5) |
| Thrombocytopenia | 1 | 0 | 1 (2) |
| Death | | 1[a] | 1 (2) |

Toxicity according to WHO criteria: 1, mild; 2, moderate; 3, severe; 4, life-threatening. Numbers are patients. [a]Sudden death on fifth day of first cycle – a causal relationship with treatment was unclear.

250 mg for constitutional symptoms caused by IFN-α was allowed. Patients were evaluated weekly for toxicity and every two cycles for response. WHO criteria for toxicity and response were used. Treatment was continued in the absence of tumour progression or severe toxicity. In the case of WHO grade 3 toxicities, treatment was withheld until complete resolution of the toxicity, and the dose of the responsible drug was reduced to 75% in subsequent cycles. Chemotherapy cycles were only restarted when WBC and platelet values were ≥ 3.5 × $10^9$ $l^{-1}$ and 100 × $10^9$ $l^{-1}$ respectively. In the case of WHO ≥ grade 3 vincristine-induced neurotoxicity or bleomycin-induced pulmonary toxicity, these drugs were permanently discontinued. A 50% dose reduction of IFN-α was allowed for ≥ grade 3 constitutional symptoms.

### RESULTS

Forty-three patients were entered onto the study in seven different centres. Patients' characteristics are listed in Table 1. All patients had stage IV disease, i.e. with distant metastases. The median age of all patients was 58 years (range 22–74), median WHO performance status 1 (0–2), median serum lactate dehydrogenase (LDH) 369 U $l^{-1}$ (161–4768, normal values up to 330 U $l^{-1}$). The median number of metastatic sites was 3 (1–5). The sites of metastases were the lungs in 24 patients, lymph nodes in 24, skin/subcutaneous in 22, liver in 17, brain in nine, bone in five and other sites in ten patients. Visceral metastases were present in 81% of patients. Seven patients had received prior systemic treatment, eight had received prior radiotherapy and nine had had surgery for metastases. Patients received a median number of two (range 1–8) cycles.

### Anti-tumour responses

Two patients were not evaluable for response. One patient died suddenly on the fifth day of treatment and one patient refused treatment after one cycle and was subsequently lost to follow-up. Thus, 41 patients were evaluable for response. The overall response rate was 27% (95% CI 14–43%) with one CR and ten PRs. No responses occurred in the seven patients who had been systemically pretreated. Of the nine patients with brain metastases,

© Cancer Research Campaign 1997

one PR occurred in a patient who had received prior cranial irradiation, whereas all four patients with asymptomatic brain metastases who were not previously irradiated progressed at this site. The response rate in 25 previously systemically untreated patients without brain metastases was 40% (95% CI 21–61%). Responses occurred in the lungs, skin/subcutaneous, lymph nodes, liver, spleen, and adrenal metastases. Median response duration was 6 months (range 2–14+ months). Median overall survival was 5 months (1–26), and in the 25 patients without prior systemic treatment and brain metastases 6 months (1–26). In four patients with progressive disease the BOLD regimen was continued, but IFN-α was given intermittently in 2-week periods interrupted by a 2-week rest period. In contrast to the original report (Pyrhönen et al, 1992), no responses or disease stabilizations were seen in these patients.

### Clinical toxicities

Forty-two patients were evaluable for toxicity. Grade 3/4 toxicity (WHO) occurred in 28 (67%) patients and consisted mainly of fatigue (33%), anorexia (14%), leucocytopenia (19%) and nausea (12%) (Table 2). A 72-year-old man with a partial response of lung metastases and a complete response of liver, subcutaneous and lymph node metastases developed pulmonary fibrosis with dyspnoea at rest after the third chemotherapy cycle. Treatment was discontinued and treatment with corticosteroids was instituted, after which his condition remained stable. He died 6 months later of brain metastases. A 59-year-old female patient with a partial response of lung metastases experienced a severe depression, which was quickly reversible after discontinuation of IFN-α. BOLD was continued for a total of six cycles. She died after 20 months of tumour progression. A 67-year-old man died suddenly at home on the fifth day of the first chemotherapy cycle before treatment with IFN-α was initiated. A tentative diagnosis of a myocardial infarction was made, and a definite causal relationship with treatment could not be established. Toxicity necessitated dose reductions or discontinuation of BOLD chemotherapy in 16 out of 134 cycles (12%) in eight patients (19%), and chemotherapy was delayed in 12 out of 134 cycles (9%) in ten patients (24%). The dose of IFN-α was reduced or discontinued in six patients (14%).

### DISCUSSION

The mechanisms underlying the supposed synergistic interaction between chemotherapy and immunotherapy are still speculative. Arguments for the enhancement of the anti-tumour activity of immunotherapy by chemotherapy as well as vice versa have been postulated. Clinical support for an interaction between these treatment modalities comes from the observation that the sequence of administration of these treatment modalities appears to be an important factor (Buzaid and Legha, 1994). Furthermore, increasing CD4/CD8 ratios during chemoimmunotherapy with BOLD plus IFN-α have been correlated with response, implying the stimulation of host defence mechanisms (Hernberg et al, 1996). The response rate with BOLD plus IFN-α in patients with metastatic melanoma in our study of 27% (95% CI 14–43%) for all patients and 40% (95% CI 21–61%) for patients without prior systemic treatment and brain metastases are lower than reported by Pyrhönen et al, (1992), who used the same doses and schedule. This difference might be explained by two factors. First, the source of IFN-α differed in the two studies. We used recombinant IFN-α-2b whereas Pyrhönen et al, used purified human leucocyte IFN-α from the Finnish Red Cross Blood Transfusion Service (Cantell et al, 1981). However, a clinical advantage for a specific type of IFN-α has never been demonstrated. Second, there are important differences between the patient populations of the two studies. In the study of Pyrhönen et al, (1992), 22 of the 48 patients (46%) had stage III disease, whereas all our patients had stage IV disease. For stage III melanoma patients with regional lymph node metastases, surgery with regional radical lymphadenectomy is the treatment of choice, and for patients with intransit metastases of an extremity isolated limb perfusion is preferred. For therapeutic groin dissections in stage III patients, 5-year survival rates of 30–47% have been reported, depending on the involvement of deep nodes (Karakousis et al, 1986). In the worst group of stage III patients, i.e. with both regional node and in-transit metastases, the 5-year survival may be 19% and the median survival time 17 months (Singletary and Balch, 1992). This is in contrast to stage IV patients with non-visceral and visceral metastases, who may have a 1-year-survival rate of 46% and 18% respectively and median survival times of 8 and 3 months respectively (Balch et al, 1992). Furthermore, 71% of our patients had a WHO performance status of ≤ 1, whereas this was the case for 92% of patients in the study by Pyrhönen et al (1992). Another aspect associated with poor prognosis was the median number of three metastatic sites in patients from our study, and in a previous review no patients with this characteristic survived for more than 1 year (Balch et al, 1992). It can therefore be concluded that our patient population consisted of a group with a worse prognosis than those in the study by Pyrhönen et al (1992). In that study, 10% of patients had brain metastases and several patients had been systemically pretreated. The median duration of response was not different between the two studies, being 6 months in our study and 6.8 months in the study of Pyrhönen et al (1992). Although tested in only four patients, we could not observe any disease regression in non-responders upon changing the IFN-α administration to an intermittent schedule (Pyrhönen et al, 1992).

The most common severe toxicity in our study was IFN-α-related malaise and fatigue, which occurred in 33% of patients. This occurred in 20% of patients in the study by Pyrhönen et al, (1992). Surprisingly, patients in this last study experienced more grade 3/4 haematological toxicity than in our study (leucocytopenia and thrombocytopenia 17% and 2% vs 32% and 11% respectively). Whether this reflects the difference in the source of IFN-α is uncertain.

In conclusion, we confirm that BOLD plus IFN-α is an active regimen in patients with metastatic melanoma. The previous reported high response rate of 62% in 48 patients is probably related to a selection of patients with good prognosis. The median survival of 6 months in this group of patients is disappointing but may be related to poor prognostic parameters.

To date, the combination of immunotherapy (IFN-α and/or IL-2) with multidrug chemotherapy (mostly including DTIC and cisplatin) has yielded the highest response rates in metastatic melanoma, with response rates up to 70% (Pyrhönen et al, 1992; Richards et al, 1992; Khayat et al, 1993; Atkins et al, 1994; Buzaid and Legha, 1994). However, all these results were obtained in (mostly single centre) phase II studies. Recently, it has been demonstrated in the setting of a randomized phase III trial in metastatic melanoma that the addition of cisplatin to a regimen of

© Cancer Research Campaign 1997

IL-2 and IFN-α significantly increases the response rate without prolonging survival (Keilholz et al, 1996). In order to provide patients with metastatic melanoma with the best possible care, further randomized phase III trials are warranted to establish the value of combination treatments vs less intensive and therefore less toxic regimens.

### ACKNOWLEDGEMENTS

The following investigators also collaborated in this study: P van Liessum, Department of Internal Medicine, Carolus Hospital's Hertogenbosch, E Balk, Department of Internal Medicine, Streekziekenhuis Gelderse Vallei Bennekom, and VA Derleyn, Department of Internal Medicine, Elkerliek Hospital Helmond, The Netherlands. The assistance in data management of M Huider, IKZ Trial Office, Eindhoven and C Terpstra, IKO Trial Office, Nijmegen, The Netherlands, is greatly appreciated. This study was financially supported by Schering Plough, The Netherlands.

### REFERENCES

Atkins MB, Oboyle KR, Sosman JA, Weiss GR, Margolin KA, Ernest ML, Kappler K, Mier JW, Sparano JA, Fisher RI, Eckardt JR, Pereira C and Aronson FR (1994) Multiinstitutional phase II trial of intensive combination chemoimmunotherapy for metastatic melanoma. *J Clin Oncol* **12**: 1553–1560

Bajetta E, Dileo A, Zampino MG, Sertoli MR, Comella G, Barduagni M, Giannotti B, Queirolo P, Tribbia G, Bernengo MG, Menichetti ET, Palmeri S, Russo A, Cristofolini M, Erbazzi A, Fowst C, Criscuolo D, Bufalino R, Zilembo N and Cascinelli N (1994) Multicenter randomized trial of dacarbazine alone or in combination with two different doses and schedules of interferon alfa-2A in the treatment of advanced melanoma. *J Clin Oncol* **12**: 806–811

Balch CM, Soong SJ, Shaw HM, Urist MM and McCarthy WH (1992) An analysis of prognostic factors in 8500 patients with cutaneous melanoma. In *Cutaneous Melanoma*, Balch CM, Houghton AN, Milton GW, Sober AJ and Soong SJ (eds), pp.165–187. JB Lippincott: Philadelphia

Buzaid AC and Legha SS (1994) Combination of chemotherapy with interleukin-2 and interferon-alfa for the treatment of advanced melanoma. *Semin Oncol* **21**: 23–28

Cantell K, Hirvonen S and Koistinen V (1981) Partial purification of human leucocyte interferon on a large scale. *Methods Enzymol* **78**: 499–505

Del Prete SA, Maurer LH, O'Donnel J, Forcier RJ and Lemarbre P (1984) Combination chemotherapy with cisplatin, carmustine, dacarbazine, and tamoxifen in metastatic melanoma. *Cancer Treat Rep* **68**: 1403–1405

Falkson CI, Falkson G and Falkson HC (1991) Improved results with the addition of interferon alfa-2b to dacarbazine in the treatment of patients with metastatic malignant melanoma. *J Clin Oncol* **9**: 1403–1408

Falkson CI, Ibrahim J, Kirkwood J and Blum R (1996) A randomized phase III trial of dacarbazine (DTIC) versus DTIC + interferon alfa-2b (IFN) versus DTIC + tamoxifen (TMX) versus DTIC + IFN + TMX in metastatic malignant melanoma: an ECOG trial (abstract). *Proc Am Soc Clin Oncol* **15**: 435

Fiedler W, Jasmin C, DE Mulder PHM, Pyrhönen S, Palmer PA, Franks CR, Oskam and Hossfeld DK (1992) A phase II study of sequential recombinant interleukin-2 followed by dacarbazine in metastatic melanoma. *Eur J Cancer* **28**: 443–446

Hernberg M, Muhonen T, Turunen JP, Hahka-Kemppinen M and Pyrhönen S (1996) The CD4 + /CD8 + ratio as a prognostic factor in patients with metastatic melanoma receiving chemoimmunotherapy. *J Clin Oncol* **14**: 1690–1696

Houghton AN, Legha S and Bajorin DF (1992) Chemotherapy for metastatic melanoma. In *Cutaneous Melanoma*, Balch CM (ed.), pp. 499–508. JB Lippincott: Philadelphia

Karakousis CP, Emrich LJ and Rao U (1986) Groin dissection in malignant melanoma. *Am J Surg* **152**: 491–497

Keilholz U, Goey SH, Punt CJA, Proebstle T, Salzmann R, Schadendorf D, Lienard D, Scheibenbogen C and Eggermont AAM (1996) A randomized trial of IFNα/IL-2 with or without CDDP in advanced melanoma: an EORTC Melanoma Cooperative Group trial (abstract). *Proc Am Soc Clin Oncol* **15**: 436

Khayat D, Borel C, Tourani JM, Benhammouda A, Antoine E, Rixe O, Vuillemin E, Bazex PA, Thill L, Franks R, Auclerc G, Soubrane C, Banzet P and Weil M (1993) Sequential chemoimmunotherapy with cisplatin, interleukin-2 and interferon alfa-2a for metastatic melanoma. *J Clin Oncol* **11**: 2173–2180

Kirkwood JM (1995) Melanoma. In *Biologic Therapy of Cancer: Principles and Practice*, DeVita VT, Jr, Hellman S and Rosenberg SA (eds), pp. 388–411 JB Lippincott: Philadelphia

Kruit WH, Punt CJA, Goey SH, Demulder PH, Vanhoogenhuyze DC, Henzenlogmans SC and Stoter G (1994) Cardiotoxicity as a dose-limiting factor in a schedule of high dose bolus therapy with interleukin-2 and alpha-interferon – An unexpectedly frequent complication. *Cancer* **74**: 2850–2856

Kruit WHJ, Punt CJA, Goey SH, DE Mulder PHM, Gratama JW, Eggermont AMM, Bolhuis RLH and Stoter G (1996) Dose-efficacy study of two schedules of high-dose bolus administration of interleukin-2 and alpha-interferon in patients with metastatic melanoma. *Br J Cancer* **74**: 951–955

Legha SS, Ring S, Papadopoulos N, Plager C, Chawla S and Benjamin R (1989) A prospective evaluation of a triple-drug regimen containing cisplatin, vinblastine, and dacarbazine (CVD) for metastatic melanoma. *Cancer* **64**: 2024–2029

McClay EF, Mastrangelo MJ, Berd D and Bellet RE (1992) Effective combination chemo/hormonal therapy for malignant melanoma: experience with three consecutive trials. *Int J Cancer* **50**: 553–556

Marincola FM, White DE, Wise AP and Rosenberg SA (1995) Combination therapy with interferon ALFA-2a and interleukin-2 for the treatment of metastatic cancer. *J Clin Oncol* **13**: 1110–1122

Marincola FM and Rosenberg SA (1995) Biologic therapy with interleukin-2: clinical applications. Melanoma. In *Biologic Therapy of Cancer*, DeVita VT, jr, Hellman S and Rosenberg SA (eds), pp. 250–262. JB Lippincott: Philadelphia

Mulder NH, Vandergraaf WTA, Willemse PHB, Koops HS, Devries EGE and Sleijfer DT (1994) Dacarbazine (DTIC)-based chemotherapy or chemoimmunotherapy of patients with disseminated malignant melanoma. *Br J Cancer* **70**: 681–683

Prudente Foundation Melanoma Study Group (1989) Chemotherapy of disseminated melanoma with bleomycin, vincristine, CCNU, and DTIC (BOLD regimen). *Cancer* **63**: 1676–1680

Pyrhönen S, Hahka-Kemppinen M and Muhonen T (1992) A promising interferon plus four-drug chemotherapy regimen for metastatic melanoma. *J Clin Oncol* **10**: 1919–1926

Richards JM, Mehta N, Ramming K and Skosey P (1992) Sequential chemoimmunotherapy in the treatment of metastatic melanoma. *J Clin Oncol* **10**: 1338–1343

Seigler HF, Lucas VS, Pickett NJ and Huang AT (1980) DTIC, CCNU, bleomycin and vincristine (BOLD) in metastatic melanoma. *Cancer* **46**: 2346–2348

Singletary SE and Balch CM (1992) Recurrent regional metastases and their management. In *Cutaneous Melanoma*, Balch CM, Houghton AN, Milton GW, Sober AJ and Soong SJ (eds), pp. 427–435. JB Lippincott: Philadelphia

Stoter G, Aamdal S, Rodenhuis S, Cleton FJ, Iacobelli S, Franks CR, Oskam R and Shiloni E (1991) Sequential administration of recombinant human interleukin-2 and dacarbazine in metastatic melanoma: a multicenter study. *J Clin Oncol* **9**: 1687–1691

Thompson DB, Adena M, McLeod GRC, Hersey P, Gill PG, Coates AS, Olver IN, Kefford RF, Lowenthal RM, Beadle GF, Walpole ET, Boland K and Kingston D (1993) Interferon-α2a does not improve response or survival when combined with dacarbazine in metastatic malignant melanoma: results of a multi-institutional Australian randomized trial. *Melanoma Res* **3**: 133–138

York M and Foltz AT (1988) Bleomycin, vincristine, lomustine, and DTIC chemotherapy for metastatic melanoma. *Cancer* **61**: 2183–2186

© Cancer Research Campaign 1997